# Loss of CD34 Expression in Aging Human Choriocapillaris Endothelial Cells

**DOI:** 10.1371/journal.pone.0086538

**Published:** 2014-01-21

**Authors:** Elliott H. Sohn, Miles J. Flamme-Wiese, S. Scott Whitmore, Kai Wang, Budd A. Tucker, Robert F. Mullins

**Affiliations:** 1 Department of Ophthalmology and Visual Sciences, The University of Iowa, Iowa City, Iowa, United States of America; 2 Stephen A. Wynn Institute for Vision Research, The University of Iowa, Iowa City, Iowa, United States of America; 3 Department of Biostatistics, The University of Iowa, Iowa City, Iowa, United States of America; Indiana University College of Medicine, United States of America

## Abstract

Structural and gene expression changes in the microvasculature of the human choroid occur during normal aging and age-related macular degeneration (AMD). In this study, we sought to determine the impact of aging and AMD on expression of the endothelial cell glycoprotein CD34. Sections from 58 human donor eyes were categorized as either young (under age 40), age-matched controls (> age 60 without AMD), or AMD affected (>age 60 with early AMD, geographic atrophy, or choroidal neovascularization). Dual labeling of sections with *Ulex europaeus* agglutinin-I lectin (UEA-I) and CD34 antibodies was performed, and the percentage of capillaries labeled with UEA-I but negative for anti-CD34 was determined. In addition, published databases of mouse and human retinal pigment epithelium-choroid were evaluated and *CD34* expression compared between young and old eyes. Immunohistochemical studies revealed that while CD34 and UEA-I were colocalized in young eyes, there was variable loss of CD34 immunoreactivity in older donor eyes. While differences between normal aging and AMD were not significant, the percentage of CD34 negative capillaries in old eyes, compared to young eyes, was highly significant (p = 3.8×10^−6^). Endothelial cells in neovascular membranes were invariably CD34 positive. Published databases show either a significant decrease in *Cd34* (mouse) or a trend toward decreased *CD34* (human) in aging. These findings suggest that UEA-I and endogenous alkaline phosphatase activity are more consistent markers of aging endothelial cells in the choroid, and suggest a possible mechanism for the increased inflammatory milieu in the aging choroid.

## Introduction

Endothelial cells (ECs) in different tissues and organs exhibit molecular heterogeneity in normal physiology to address tissue-specific needs [1], [2]. This EC specialization includes highly variable characteristics with respect to degree of permeability, response to vascular endothelial growth factors, synthesis of hemostasis mediators, and leukocyte recruitment/expression of cluster differentiation (CD) antigens [2]–[5]. Rather than being fixed at birth, ECs retain a high degree of plasticity, altering their properties in response to the needs of the organism and based on microenvironmental changes. [2], [6] In normal aging and in diseases of the vasculature, the phenotype of ECs and their ability to respond to microenvironmental stimuli becomes impaired (see for example [7], [8]).

Age-related macular degeneration (AMD) is characterized in part by increased leukocytes in the choroid, the vascular bed supplying the outer neural retina and retinal pigment epithelium [9]–[11]. A more thorough understanding of the behavior of proteins that mediate migration of leukocytes into the choroid is therefore likely to provide insight into the pathogenesis of AMD. Leukocyte transmigration is mediated by both soluble mediators and cell surface proteins, both of which have been studied in aging eyes and eyes with AMD [12]–[16].

In a previous screen of endothelial cell adhesion markers in eyes with AMD, we noted that some capillaries that were reactive with the lectin *Ulex europaeus* agglutinin-I (UEA-I), previously used as a marker of viable endothelial cells [11], did not exhibit immunoreactivity with antibodies directed against CD34, a cell surface glycoprotein expressed on vascular endothelial cells. In the current report, we quantified this loss of endothelial CD34 expression as a function of age and different phenotypes of AMD. Our findings support the idea that aging choriocapillaris endothelial cells undergo loss of CD34 expression.

## Methods

Human donor eyes were obtained from the Iowa Lions Eye Bank following informed consent of the donors’ next of kin. All experiments were performed in accordance with the Declaration of Helsinki. Maculae used in this study were preserved by fixation in 4% paraformaldehyde diluted in 10mM phosphate buffered saline (PBS) for 2 hours, and were subsequently cryoprotected and embedded in sucrose solution as described previously by Barthold and Raymond [17].

Clinical diagnosis of AMD was determined by chart review and, for advanced AMD, confirmed by histological analysis. Fifty-eight eyes from 54 donors were studied. Samples evaluated included 18 eyes from 17 unaffected controls (average age  = 81.7, range 60–91); 17 eyes with early/dry (non-atrophic, non-neovascular) AMD (average age  = 87.4, range 69–100); 5 eyes with neovascular AMD (average age  = 80.8, range 77–91), 8 eyes with geographic atrophy (average age  = 84.4, range 78–96), and 10 “young” eyes (average age  = 30.1, range 5 to 45).

For immunohistochemical experiments, frozen sections were dual labeled with anti-CD34 (NeoMarkers and Thermo Scientific Pierce) and the fucose binding lectin, *Ulex europaeus* agglutinin- I (UEA-I). Briefly, sections were blocked in 1mg/mL bovine serum albumin, followed by simultaneous incubation of sections with anti-CD34 (1∶250) and biotinylated UEA-I (Vector Laboratories, Burlingame, CA) diluted 1∶50. After 1 hour of incubation, sections were washed and incubated with Alexa-488-conjugated anti-mouse IgG (Invitrogen, Eugene, OR) and 1∶100 avidin-Texas red (Vector), with 100 µg/mL diamidino-phenyl-indole (DAPI, Sigma). After 30 minutes incubation, sections were washed and coverslipped. Negative controls included omission of primary antibody. For all incubations and washes, buffers included 1mM each of MgCl_2_ and CaCl_2_.

Sections were photographed using an epifluorescence microscope (Olympus BX41) with a digital camera (SPOT-RT; Diagnostic Instruments). Individual grayscale images were taken using each filter set and RGB images were created by merging individual channels.

In 3 eyes that showed some loss of CD34 expression, we sought to identify the relationship between UEA-I labeling, CD34 expression, and endogenous alkaline phosphatase, a histochemical feature of choroidal endothelial cells [18], [19]. Sections were dual labeled as above, except that they were coverslipped only in PBS. Sections were photographed immediately and coverslips carefully removed, followed by incubation with NBT/BCIP solution (Vector) in Tris-HCl buffer pH 9, according to the manufacturer’s instructions. After re-coverslipping, care was taken to collect color photomicrographs from the same regions of the macula previously imaged for UEA-I/CD34.

For quantification of CD34(−) capillaries, photomicrographs were evaluated in ImageJ software [20] and a masked observer identified capillaries as UEA-I(+)/CD34(+) or UEA-I(+)/CD34(−). A total of 3,078 capillaries were scored, with an average of 53.9 capillaries scored per donor. Samples were then unmasked as to AMD affection phenotype. Logistic regression analysis was performed on the association between CD34 loss and risk factors (age and AMD state) using the R statistical software (R Core Team, 2013).

In order to assess the expression of *CD34* in published datasets of ocular gene expression, Agilent Whole Human Genome 4×44 K in situ oligonucleotide microarray data described by Newman and colleagues [21] was downloaded from NCBI’s Gene Expression Omnibus (GEO; accession GSE29801). RPE-choroid samples were quantile normalized using the ‘normalizeBetweenArrays’ function available in the ‘limma’ package [22] for the R statistical software (R Core Team, 2013). Arrays from donors less than 60 years of age (n = 15) were compared to arrays from donors 60 years or older (n = 35); all samples were normal with respect to AMD diagnosis. Differential expression was assessed using a moderated t-test implemented in the ‘limma’ package.

In addition, Affymetrix Mouse 430 2.0 microarray data described by Chen and colleagues [23] was downloaded from NCBI’s GEO (accession GSE10965). CEL files were normalized using the Robust Multi-array Average (RMA) algorithm as implemented in the ‘affy’ package [24] for R. RPE/choroid tissue from four month old male C57BL/6 mice (n = 4) were compared to that of 26 month old male C57BL/6 mice (n = 4). Differential expression was assessed using a moderated t-test implemented in the ‘limma’ package.

## Results

**Figure 1:**
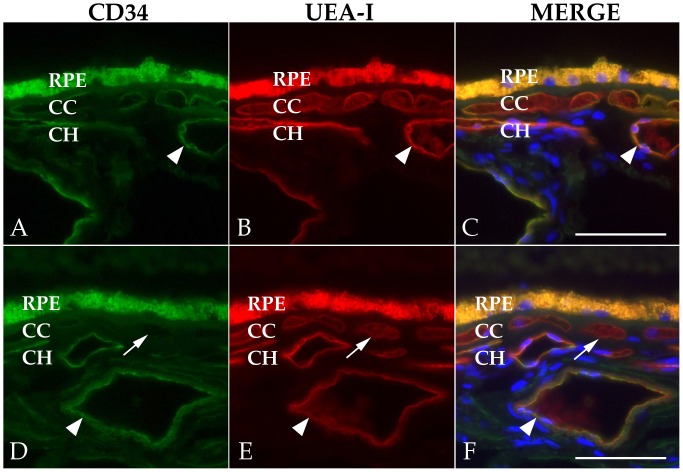
Loss of CD34 in a subset of aging capillaries. Eyes from two donors are depicted, ages 85 (A–C) and 88 (D–F). Large vessel endothelial cells showed retention of CD34 expression. Arrows in D–F indicate choriocapillaris vessels with greatly reduced CD34 reactivity. Large vessels (arrowheads) retained expression. Green fluorescence, anti-CD34 labeling; red fluorescence, UEA-I; blue fluorescence, DAPI. Scalebar = 50 µm.

Observations on donor eyes from elderly individuals revealed molecular heterogeneity of expression at the level of the choriocapillaris, with occasional vessels that stained with the lectin UEA-I, but did not label with antibodies directed against CD34, a marker used frequently in other studies to identify choroidal endothelial cells (**Figure 1**). The same eyes that showed loss of choriocapillaris CD34 exhibited labeling of large vessel endothelial cells and solitary cells in the choroid and sclera, making it unlikely that this loss in the choriocapillaris was artifactual. This observation led us to question whether this capillary phenotype was characteristic of eyes with advancing age and/or AMD.

**Figure 2:**
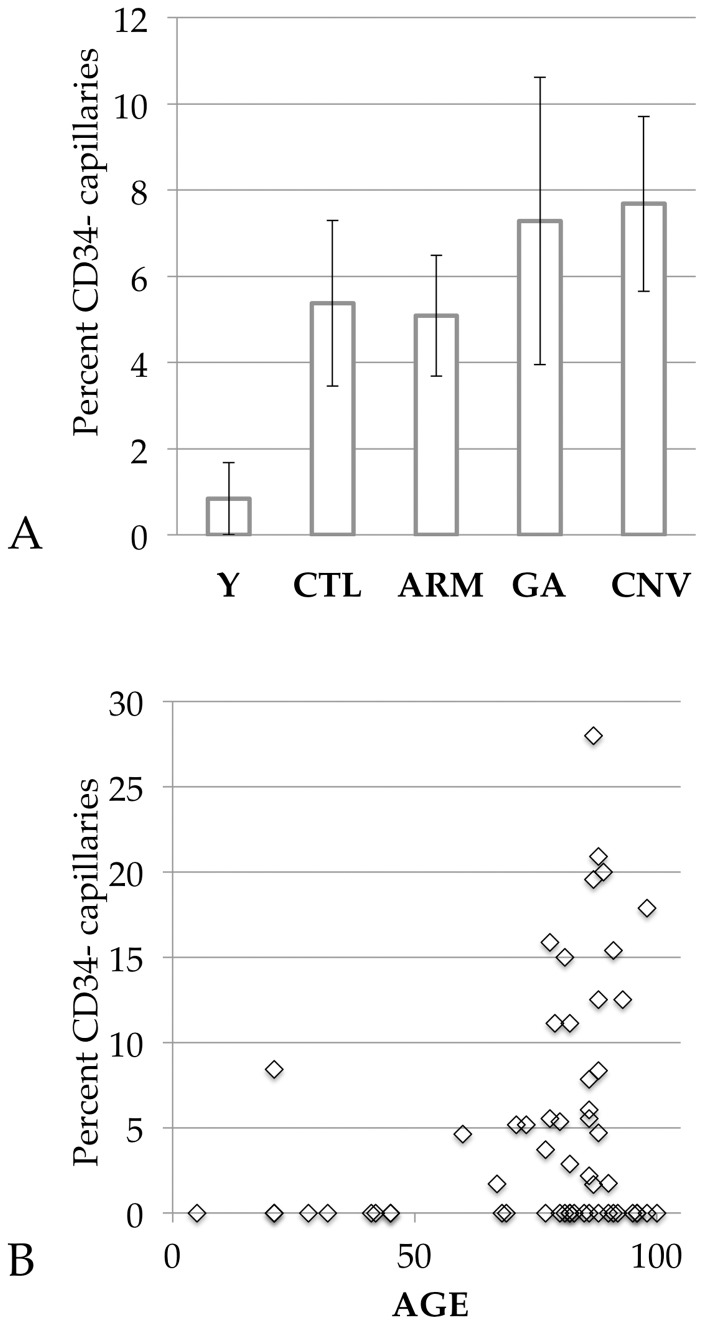
Percentage of CD34(−) capillaries in human choriocapillaris. (A) Average values segregated by disease phenotype. Y = young (mean age  = 31.1); CTL = age-matched controls; ARM = early/dry AMD; GA = geographic atrophy; CNV = choroidal neovascularization. Error bars indicate the standard error of the mean. (B) All donors plotted as percent capillaries without CD34 versus age. Note the age related increase in CD34(−) capillaries (p<10^−5^).

We addressed this question by identifying a series of donor eyes from older individuals with different stages of AMD. The percentage of CD34(−) capillaries did not differ significantly between age-matched controls and any phenotype of AMD (**Figure 2A**) (p>0.05); however, in eyes with geographic atrophy, this number approached statistical significance (p = 0.06).

We then speculated that age might be a factor in loss of CD34 expression. We evaluated a series of 10 donors at or under the age of 45. In all but one donor in this set, 100% of the UEA-I(+) capillaries were also CD34(+). In one sample, a significant proportion of capillaries (8.4%) lacked CD34 expression. There was no obvious difference between this donor (a 21 year old female, cause of death Hodgkin’s lymphoma) and the rest of the young cohort, nor any plausible justification to disqualify this sample from the analysis.

**Figure 3:**
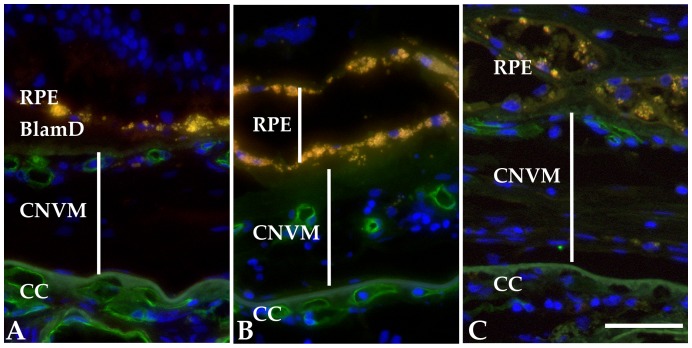
Expression of CD34 in pathologic blood vessels in choroidal neovascular membranes (CNVMs) from 3 donors with neovascular AMD. Abnormal vessels within the CNVMs were invariably strongly positive (arrows). RPE, dystrophic retinal pigment epithelium; BlamD, layer of basal laminar deposit; CC, choriocapillaris. Scalebar in C, 50 µm.

Comparison of all samples in both young and old age groups reveals a very strong association with CD34 loss in aging choriocapillaris endothelial cells. The average percentage of CD34(−) capillaries in all young donors was 0.84%, compared to 5.8% in the older group. This age-related decrease in CD34(+) capillaries was highly significant (p = 3.8×10^−6^)(**Figure 2B**). In the subset of eyes with choroidal neovascularization, ECs within the CNVM were invariably positive (**Figure 3**).

**Figure 4:**
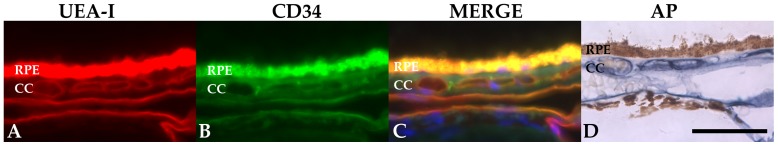
Tandem triple labeling of CD34/UEA-I and endogenous alkaline phosphatase. Following epifluorescence photomicrography (A, B,C), coverslips were removed and sections reacted with NBT/BCIP. Note the good correspondence between alkaline phosphatase activity (D) and UEA-I binding (A) that includes capillaries that do not react with CD34 antibody (B). CC, choriocapillaris; scalebar = 50 µm.

We then sought to determine whether loss of CD34 expression was generalizable to other EC markers and represented a general age-related EC modifications. In experiments in which the relationship between CD34, UEA-I and endogenous alkaline phosphatase was examined, UEA-I(+) capillaries that were unlabeled by anti-CD34 antibodies showed persistent expression of endogenous alkaline phosphatase (**Figure 4**). While not exhaustive, this experiment indicates better overlap of UEA-I and endogenous alkaline phosphatase than either marker with anti-CD34 antibody.

Analysis of published gene expression datasets showed some loss of choroidal *CD34* expression at the transcriptional level. In a large microarray experiment using human samples of RPE-choroid, Newman et al. included a young (<60 years of age) cohort [21]. *CD34* expression in the young group (7.48±0.85) was approximately 35% higher than in the >60 years of age non-AMD group (7.05±0.88); however, this difference did not reach statistical significance (p = 0.095). Similarly, comparing the RPE-choroid layer of young and old mice from the study performed by Chen et al. [23] shows that expression of *Cd34* in young mice was about 47% higher than that of old mice (raw p = value <.0005, adjusted p value <0.01).

## Discussion

Cluster differentiation antigen 34 (CD34) is a single pass, transmembrane glycoprotein. Similar to its paralogs, podocalyxin and endoglycan, CD34 is highly O-glycosylated sialoprotein with roles in endothelial cell-leukocyte interactions. While predominately expressed in the vasculature in the human eye, CD34 is a widespread marker of hematopoietic cells [25].

We have previously suggested that, in evaluating health of vascular beds in donor specimens, using a robust marker for viable EC is essential to avoid misidentifying vessels that are still present as “ghost” capillaries [11]. We saw similar patterns of distribution in young donors between CD34 and UEA-I, but noted that in older donors there was significant, and capillary-specific, loss of CD34 expression. UEA-I and alkaline phosphatase cytochemistry were similar in eyes with loss of CD34 expression. CD34 immunoreactivity is frequently used to visualize choroidal ECs. While this marker appears consistently expressed in very young eyes [26], we suggest that, in aging eyes, UEA-I and endogenous alkaline phosphatase are more stable markers of endothelial cell viability than CD34.

On the other hand, loss of CD34 expression in aging maculae may have biological consequences that are relevant to the pathogenesis of AMD or other macular diseases. While we did not observe a compelling AMD-associated phenotype, with only geographic atrophy eyes approaching statistical significance on linear regression, age is the strongest risk factor for AMD, and aging changes in the choriocapillaris may provide insights into the conditions permissive for the development of AMD.

While normally considered an adhesion molecule, CD34 plays complex and counterintuitive roles in leukocyte-endothelial interactions. Like other sialomucins in the same family (endoglycan and podocalyxin), CD34 is highly glycosylated. However, only in high endothelial venules does CD34 possess a sialyl-Lewis-X antigen glycosylation profile that serves as a ligand for leukocyte recruitment via L-selectin-carbohydrate interactions [27]. Instead, when lacking the Lewis antigen (i.e., in most of the vasculature) CD34 is believed to inhibit leukocyte-endothelial cell binding, due to the highly negatively charged sialic acid residues, in a mechanism referred to as “molecular Teflon”. [27].

If CD34 is similarly anti-adhesive in choroidal endothelial cells, the age-related loss of CD34 expression could result in increased leukocyte trafficking in aging eyes. Increased numbers of CD68+ macrophages have been observed in Bruch’s membrane in aging eyes, particularly with extensive subRPE deposits. [28] Since leukocytes have a variety of potential helpful and harmful functions in the aging macula [29] [30] [31], an age-related increase in leukocyte transmigration could impact the health of the macula and contribute to the pathophysiology of AMD.

Limitations of this study include the relatively non-quantitative measurement of CD34 negative capillaries. Since loss of CD34 expression is likely to occur along a spectrum, only capillaries with severe loss of CD34 were counted as negative. However, consistent with our human findings, assessment of gene expression in aged mouse RPE-choroid reveals a significant reduction of *Cd34* mRNA. Similarly, studies by Newman et al. on human RPE-choroid show a trend toward decreased expression in the older, compared to the <60 group, however this difference did not reach statistical significance. These differences may reflect: (a) the molecular differences between human and mouse; (b) the persistent expression of CD34 in most choroidal EC in human aging, diluting the effects of loss of expression in subsets of ECs; and/or (c) the fact that genetically identical, inbred mice, may reveal straightforward effects of age compared to humans in which inter-individual difference in environment, cause of death, and genetic variability complicates molecular analyses.

In summary, we identified a loss of immunologically detectable CD34 in aging human choroiocapillaris ECs. These findings suggest that the detection of CD34 as a marker of human choriocapillaris EC is likely to be robust and comprehensive in young eyes, but in advanced age can result in incorrectly identifying a capillary EC as dead when it has instead changed its differentiation state. In these older specimens, detection of endogenous alkaline phosphatase or fucose moieties with UEA-I is more reliable. Moreover, the loss of CD34 suggests a possible mechanism for an age-related increase in leukocyte trafficking into the choroid.

## References

[1] Molema G, Aird WC (2012) Vascular heterogeneity in the kidney. Semin Nephrol 32: 145–155 10.1016/j.semnephrol.2012.02.001 22617763

[2] Aird WC (2007) Phenotypic heterogeneity of the endothelium: I. Structure, function, and mechanisms. Circ Res 100: 158–173 10.1161/01.RES.0000255691.76142.4a 17272818

[3] McLeod DS, Lefer DJ, Merges C, Lutty GA (1995) Enhanced expression of intracellular adhesion molecule-1 and P-selectin in the diabetic human retina and choroid. Am J Pathol 147: 642–653. 7545873 PMC1870979

[4] Michel CC, Curry FE (1999) Microvascular permeability. Physiol Rev 79: 703–761. 10390517 10.1152/physrev.1999.79.3.703

[5] Mackman N (2005) Tissue-specific hemostasis in mice. Arterioscler Thromb Vasc Biol 25: 2273–2281 10.1161/01.ATV.0000183884.06371.52 16123318

[6] Roberts WG, Palade GE (1995) Increased microvascular permeability and endothelial fenestration induced by vascular endothelial growth factor. J Cell Sci 108 (Pt 6): 2369–2379. 10.1242/jcs.108.6.2369 7673356

[7] Klement H, St Croix B, Milsom C, May L, Guo Q, et al (2007) Atherosclerosis and vascular aging as modifiers of tumor progression, angiogenesis, and responsiveness to therapy. Am J Pathol 171: 1342–1351 10.2353/ajpath.2007.070298 17823292 PMC1988883

[8] Erusalimsky JD (2009) Vascular endothelial senescence: from mechanisms to pathophysiology. J Appl Physiol 106: 326–332 10.1152/japplphysiol.91353.2008 19036896 PMC2636933

[9] Penfold PL, Killingsworth MC, Sarks SH (1985) Senile macular degeneration: the involvement of immunocompetent cells. Graefes Arch Clin Exp Ophthalmol 223: 69–76. 2408968 10.1007/BF02150948

[10] Ezzat MK, Hann CR, Vuk-Pavlovic S, Pulido JS (2008) Immune cells in the human choroid. Br J Ophthalmol 92: 976–980 10.1136/bjo.2007.129742 18577650

[11] Mullins RF, Johnson MN, Faidley EA, Skeie JM, Huang J (2011) Choriocapillaris vascular dropout related to density of drusen in human eyes with early age-related macular degeneration. Invest Ophthalmol Vis Sci 52: 1606–1612 10.1167/iovs.106476 21398287 PMC3101687

[12] Wang H, Wittchen ES, Jiang Y, Ambati B, Grossniklaus HE, et al (2011) Upregulation of CCR3 by age-related stresses promotes choroidal endothelial cell migration via VEGF-dependent and -independent signaling. Invest Ophthalmol Vis Sci 52: 8271–8277 10.1167/iovs.118230 21917937 PMC3208059

[13] Takeda A, Baffi JZ, Kleinman ME, Cho WG, Nozaki M, et al (2009) CCR3 is a target for age-related macular degeneration diagnosis and therapy. Nature 460: 225–230 10.1038/nature08151 19525930 PMC2712122

[14] Yeh DC, Bula DV, Miller JW, Gragoudas ES, Arroyo JG (2004) Expression of leukocyte adhesion molecules in human subfoveal choroidal neovascular membranes treated with and without photodynamic therapy. Invest Ophthalmol Vis Sci 45: 2368–2373. 15223819 10.1167/iovs.03-0981

[15] Bhutto IA, McLeod DS, Merges C, Hasegawa T, Lutty GA (2006) Localisation of SDF-1 and its receptor CXCR4 in retina and choroid of aged human eyes and in eyes with age related macular degeneration. Br J Ophthalmol 90: 906–910 10.1136/bjo.2006.090357 16597663 PMC1857162

[16] Skeie JM, Fingert JH, Russell SR, Stone EM, Mullins RF (2010) Complement component C5a activates ICAM-1 expression on human choroidal endothelial cells. Invest Ophthalmol Vis Sci 51: 5336–5342 10.1167/iovs.105322 20484595 PMC3066598

[17] Barthel LK, Raymond PA (1990) Improved method for obtaining 3-microns cryosections for immunocytochemistry. J Histochem Cytochem 38: 1383–1388. 2201738 10.1177/38.9.2201738

[18] Lutty GA, McLeod DS (2005) Phosphatase enzyme histochemistry for studying vascular hierarchy, pathology, and endothelial cell dysfunction in retina and choroid. Vision Res 45: 3504–3511 10.1016/j.visres.2005.08.022 16213000 PMC4928484

[19] McLeod DS, Grebe R, Bhutto I, Merges C, Baba T, et al (2009) Relationship between RPE and choriocapillaris in age-related macular degeneration. Invest Ophthalmol Vis Sci 50: 4982–4991 10.1167/iovs.093639 19357355 PMC4829357

[20] Abramoff MD, Magalhaes PJ, Ram SJ (2004) Image Processing with ImageJ. Biophotonics International 11: 36–42.

[21] Newman AM, Gallo NB, Hancox LS, Miller NJ, Radeke CM, et al (2012) Systems-level analysis of age-related macular degeneration reveals global biomarkers and phenotype-specific functional networks. Genome Med 4: 16 10.1186/gm315 22364233 PMC3372225

[22] Smyth GK (2005) Limma: linear models for microarray data. Gentleman R, Carey V, Dudoit S, Irizarry R, Huber W, editors New York: Springer. 24 pp.

[23] Chen H, Liu B, Lukas TJ, Neufeld AH (2008) The aged retinal pigment epithelium/choroid: a potential substratum for the pathogenesis of age-related macular degeneration. PLoS ONE 3: e2339 10.1371/journal.pone.0002339 18523633 PMC2394659

[24] Gautier L, Cope L, Bolstad BM, Irizarry RA (2004) affy–analysis of Affymetrix GeneChip data at the probe level. Bioinformatics 20: 307–315 10.1093/bioinformatics/btg405 14960456

[25] Steen R, Egeland T (1998) CD34 molecule epitope distribution on cells of haematopoietic origin. Leuk Lymphoma 30: 23–30 10.3109/10428199809050926 9669673

[26] Lutty GA, Hasegawa T, Baba T, Grebe R, Bhutto I, et al (2010) Development of the human choriocapillaris. Eye 24: 408–415 10.1038/eye.2009.318 20075975 PMC4848024

[27] Nielsen JS, McNagny KM (2008) Novel functions of the CD34 family. J Cell Sci 121: 3683–3692 10.1242/jcs.037507 18987355

[28] Cherepanoff S, McMenamin P, Gillies MC, Kettle E, Sarks SH (2010) Bruch’s membrane and choroidal macrophages in early and advanced age-related macular degeneration. Br J Ophthalmol 94: 918–925 10.1136/bjo.2009.165563 19965817

[29] Skeie JM, Mullins RF (2009) Macrophages in neovascular age-related macular degeneration: friends or foes? Eye 23: 747–755 10.1038/eye.2008.206 18600240 PMC8204908

[30] Sene A, Khan AA, Cox D, Nakamura REI, Santeford A, et al (2013) Impaired cholesterol efflux in senescent macrophages promotes age-related macular degeneration. Cell Metab 17: 549–561 10.1016/j.cmet.2013.03.009 23562078 PMC3640261

[31] Grossniklaus HE, Ling JX, Wallace TM, Dithmar S, Lawson DH, et al (2002) Macrophage and retinal pigment epithelium expression of angiogenic cytokines in choroidal neovascularization. Mol Vis 8: 119–126. 11979237

